# Alpha oscillations do not implement gain control in early visual cortex but rather gating in parieto‐occipital regions

**DOI:** 10.1002/hbm.25183

**Published:** 2020-08-21

**Authors:** Alexander Zhigalov, Ole Jensen

**Affiliations:** ^1^ Centre for Human Brain Health, School of Psychology University of Birmingham Birmingham UK

**Keywords:** alpha oscillations, frequency tagging, magnetoencephalography, response latency, spatial attention

## Abstract

Spatial attention provides a mechanism for, respectively, enhancing relevant and suppressing irrelevant information. While it is well established that attention modulates oscillations in the alpha band, it remains unclear if alpha oscillations are involved in directly modulating the neuronal excitability associated with the allocation of spatial attention. In this study, in humans, we utilized a novel broadband frequency (60–70 Hz) tagging paradigm to quantify neuronal excitability in relation to alpha oscillations in a spatial attention paradigm. We used magnetoencephalography to characterize ongoing brain activity as it allows for localizing the sources of both the alpha and frequency tagging responses. We found that attentional modulation of alpha power and the frequency tagging response are uncorrelated over trials. Importantly, the neuronal sources of the tagging response were localized in early visual cortex (V1) whereas the sources of the alpha activity were identified around parieto‐occipital sulcus. Moreover, we found that attention did not modulate the latency of the frequency tagged responses. Our findings point to alpha band oscillations serving a downstream gating role rather than implementing gain control of excitability in early visual regions.

## Graphical abstract

By combining magnetoencephalography and a novel broadband frequency tagging approach, we show that spatial attention differently modulates alpha oscillations and neuronal excitability. Importantly, the sources of the alpha oscillations and tagging responses were spatially distinct and the alpha power and tagging response were not related over trials. These results are inconsistent with previous ideas suggesting that alpha oscillations are involved in gain control of early sensory regions; rather alpha oscillations are involved in the allocation of neuronal resources in downstream regions.

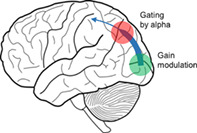

## 1 INTRODUCTION

Attention provides a mechanism that allows enhancing relevant and suppressing irrelevant information (Kastner & Nobre, 2014). When several complex stimuli are presented in a visuospatial scene, a selection mechanism is enhancing and suppressing relevant and irrelevant information, respectively. Attention results in competitive interactions among neurons, causing them to respond stronger to attended stimuli while distracting stimuli are suppressed (Desimone & Duncan, 1995). The underlying neuronal mechanisms of these modulations in relation to oscillatory brain activity remain elusive. Several studies showed that the power of alpha band oscillations is modulated by attention (Foxe & Snyder, 2011; Jensen & Hanslmayr, 2020; Klimesch, 2012) and it has been suggested that alpha oscillations modulates gain control mechanism by influencing neuronal excitability (Jensen & Mazaheri, 2010; Mathewson et al., 2011; Romei et al., 2008; van Diepen, Foxe, & Mazaheri, 2019). It is also known that attention modulates power of neuronal response to flickering (or tagging) stimuli at higher frequencies (Gulbinaite, Roozendaal, & VanRullen, 2019). Interestingly, while alpha power decreases contralaterally to attended stimulus (and increases ipsilaterally), power of the high‐frequency tagging response shows an opposite effect (e.g., Gulbinaite et al., 2019; Zhigalov, Herring, Herpers, Bergmann, & Jensen, 2019). There have been some attempts to assess the relationship between power of the alpha oscillations and the tagging response in attentional tasks. These studies indicate that the frequency tagging signal is not related to alpha magnitude on a trial‐by‐trial basis (Gundlach, Moratti, Forschack, & Müller, 2020; Zhigalov et al., 2019). This then begs the question of what is the functional role of alpha oscillations in attention tasks? Magnetoencephalography (MEG) offers the opportunity to localize the sources of the alpha oscillations and the frequency tagging response. If the sources are at different levels of the visual hierarchy, this would provide important clues to the functional role of alpha oscillations.

Broadband frequency tagging also allows us to assess the delay of the neuronal response with respect to visual input. While it is well established that spatial attention can increase neuronal responses, it might also speed up processing, that is, neurons in visual cortex responding to attended objects will fire earlier compared to those for unattended objects. A study by Sundberg, Mitchell, Gawne, and Reynolds (2012) showed that attention does produce small (1–2 ms) but significant reductions in the latency of both the spiking and LFP responses in extrastriate cortex V4. Similarly, another study demonstrated that both latency and peak amplitude of the response are modulated by attention, but only latencies correlate with reaction time (Galashan, Saßen, Kreiter, & Wegener, 2013). These findings motivate exploring if response latencies change with attention in humans. As we will explain, processing delays can be estimated by cross correlating the broadband visual input with the MEG response.

In this study, we used a novel experimental design to dissociate the effect of attention on the alpha and tagging responses. We utilized a spatial attention task where the luminance of visual stimuli in the left and right visual field was driven by independent random broadband signals (60–70 Hz). This broadband frequency tagging technique in combination with MEG allowed us to obtain reliable responses to invisible flicker that did not perturb the alpha oscillations, and also estimate response latencies by cross correlating the tagging signal and the brain response as a function of attention. This allows us to correlate the frequency tagged response and alpha power over trials. We applied source modeling to localize the neuronal activity associated with the alpha and tagging responses.

## 2 MATERIALS AND METHODS

### 2.1 Participants

Twenty‐four right‐handed participants (mean age: 31; age range: 23–38; 14 females) with no history of neurological disorders partook in the study. Six of the participants (four females) were excluded from the analysis: two of the participants had noisy MEG signal due to electronics failure, while four other participants showed an excessive amount of motion and muscle artifacts, eye blinks and saccades so that the remaining trials did not allow us to reliably assess power and latency of the tagging response. This left 18 participants for further analysis.

The study was approved by the local ethics committee (University of Birmingham, UK) and written informed consent was acquired before enrolment in the study. All participants conformed to standard inclusion criteria for MEG experiments. Participants had normal or corrected‐to‐normal vision. Participants received financial compensation of £15 per hour or were compensated in course credits.

### 2.2 Experimental paradigm

**FIGURE 1:**
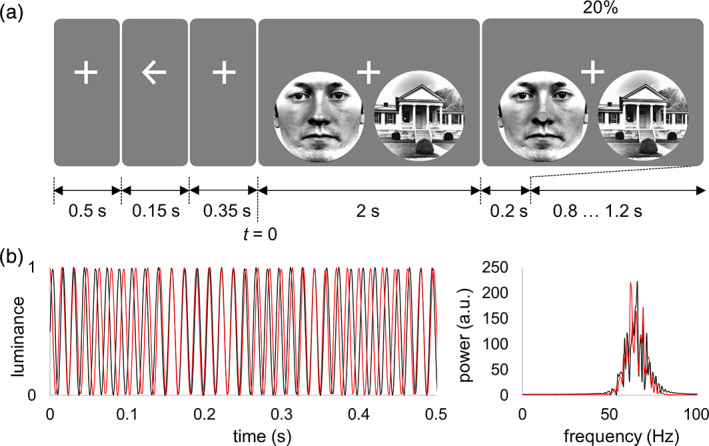
(a) The experimental paradigm. After a cue (left/right arrow), a house‐face pair was presented superimposed with broadband (60–70 Hz) flicker signals. In 20% of the trials, the target stimulus (i.e., a small circle of 1.2° vertical translation around the center of the object) occurred on the cued side and required participant's response. In 5% of the trials (catch trials), the target was presented in the hemifield opposite to the cued side and participants had to ignore this event. The onset of frequency tagging is marked as *t* = 0. (b) Example of the two uncorrelated broadband signals (red and black lines depict left and right stimuli, respectively) used to drive the visual stimuli and the respective power spectra. Note that the broadband signals were generated independently for each trial

Participants performed a spatial attention task (eight blocks of 6 min) in which they were instructed to allocate attention to either the left or right visual hemifield in accordance with the cue presented at the beginning of each trial (Figure 1). Each trial started with a fixation cross (500 ms) followed by a cue (150 ms; left/right arrow) indicating the hemifield that the participants had to attend to, while fixating on the center of the screen. A fixation cross was shown for 350 ms after the attentional cue, and then stimuli were presented in the left and right visual hemifield for 2,000 ms. Participants were instructed to detect a target stimulus (a small circle) occurring at the center of the stimulus (i.e., face or house). The target stimulus occurred at the end of the trial in 25% of the trials. In 20% of the trials, the target was presented on the cued side, while in 5% of the trials (catch trials) the target was on uncued side to which participants did not have to respond. Participants responded to the target by pressing a button by either left or right index finger (counterbalanced over participants). The duration of the target was adjusted using QUEST adaptive staircase procedure (Watson & Pelli, 1983) to attain 80% correct responses. The initial duration of the target was 10 ms and it varied between 2 and 30 ms during the session controlled by the QUEST procedure. The validity of the responses was indicated on the screen as correct (“CORRECT”), incorrect (“INCORRECT”), or missed (“MISS”) response. The next trial started after a random interval of 800 ± 200 ms. Such relatively short inter‐stimulus interval may influence the neuronal responses in the subsequent trials; however, the random stimulus onset reduces this effect. The experimental paradigm was implemented in MATLAB 2018b (MathWorks Inc., Natick, MA) using Psychophysics Toolbox 3.0.11 (Kleiner et al., 2007).

### 2.3 Visual stimuli

Participants were seated upright in the MEG scanner in front of the screen (1.5 m) and pairs of rounded stimuli (face and house; 5.7° wide) were presented simultaneously in the lower left and right visual field to enhance the occipital cortical response (Portin, Vanni, Virsu, & Hari, 1999). The centers of the left and right stimuli were placed a 3.8° eccentricity. The target stimulus to which participants were instructed to respond was implemented as a small circle of 1.2° vertical translation around the center of the object (i.e., face or house). Different combinations of faces and houses (comprising 10 faces and 10 houses) were presented in random order over the trials. The luminance of the grayscale stimuli was normalized using the SHINE Toolbox for MATLAB (Willenbockel et al., 2010) (see Figure 1).

The luminance of the stimuli (face and house) was modulated by broadband signals (60–70 Hz). To avoid phase discontinuity, the broadband tagging signal (Figure 1b) was generated using phase modulation as follows

(1) $$S=\sin\left(2\mathrm{\pi t}f_{c}+\sum_{i=1}^{3}\sin\left(2\mathrm{\pi t}\left(i+r_{i}/4+1\right)\right)\right)$$

where *f*_*c*_ denotes frequency of the carrier signal (65 Hz), *t* is a time variable (0–2 s), and *r*_*i*_ is a random number from uniform distribution [0, 1]. The tagging signals for the left and right stimuli were generated independently for each trial, and these signals were uncorrelated in the 2 s interval, that is, the correlation was below 0.1. Direction of attention, pairing of face‐house stimuli, and tagging frequencies were counterbalanced over trials.

### 2.4 Projector

We used the PROPixx DLP LED projector (VPixx Technologies Inc., Canada) to present the visual stimuli. This projector provides a refresh rate up to 1,440 Hz by dividing each frame received from the graphics card (at 120 Hz) into multiple frames. The projector divides each received frame (1,920 × 1,200 pixels) into four equally sized quadrants (960 × 600 pixels), allowing for a fourfold increase in refresh rate (480 Hz). Color (RGB) images presented in each quadrant are further converted to a grayscale representation by equalizing all components of RGB code. This allows for a 1,440 Hz refresh rate since the 120 Hz is multiplied by a factor of, respectively, fourfold and threefold when presenting grayscale images with a resolution of 960 × 600 pixels.

### 2.5 MEG and MRI data acquisition

MEG was acquired using a 306‐sensor TRIUX Elekta Neuromag system (Elekta, Finland). The MEG data were low‐pass filtered at 300 Hz using embedded anti‐aliasing filters and sampled at 1,000 Hz. Head position of the participants was digitized using the Polhemus Fastrack electromagnetic digitizer system (Polhemus Inc.). We also used an EyeLink eye tracker, and vertical and horizontal EOG sensors to remove trials containing blinks and saccades.

The tagging signals were recorded using a custom‐made photodetector (Aalto NeuroImaging Centre, Aalto University, Finland) that was connected to a miscellaneous channel of MEG system. This allowed us to acquire the tagging signal with the same temporal precision as the MEG data.

A high‐resolution T1‐weighted anatomical image (TR/TE of 7.4/3.5 ms, a flip angle of 7°, FOV of 256 × 256 × 176 mm, 176 sagittal slices, and a voxel size of 1 × 1 × 1 mm^3^) was acquired using 3‐T Phillips Achieva scanner.

### 2.6 MEG data preprocessing

MEG data were analyzed using MATLAB and the Fieldtrip toolbox (Oostenveld, Fries, Maris, & Schoffelen, 2011). The data were segmented into 3.5 s epochs; −1.0 to 2.5 s relative to the onset of the flickering stimuli (houses and faces).

Eye blinks were detected in the X‐axis and Y‐axis channels of the eye tracker by applying a threshold of 5 SD. The saccades were detected using scatter diagram (or joint histogram) of X‐axis and Y‐axis time series of the eye tracker for each trial. An event was classified as a saccade if the focus away from the central cross (i.e., fixation point) lasted longer than 500 ms. The trials contaminated by blinks and saccades were removed from further analysis. We also rejected trials containing large amplitude events (above 5 SD) in MEG which are mainly associated with motion and muscle artifacts. As a result, the number of trials that survived all the exclusion criteria was 534 ± 38 (mean ± SD) per participant, and for each participant, the number of trials per condition was equalized by randomly selecting the same number of trials.

### 2.7 Attention modulation index

We quantified the effect on attention on the power at the alpha power and tagging signal using attention modulation index (AMI). To this end, spectral power was computed using Fourier transform for each MEG sensor and each epoch from 0 to 2 s relatively to stimulus onset:

(2) $$\mathrm{AMI}=\left(P_{\text{attended}}-P_{\text{unattended}}\right)/\left(P_{\text{attended}}+P_{\text{unattended}}\right)$$

where *P*_attended_ and *P*_unattended_ denote spectral power averaged over attended and unattended stimuli trials, respectively.

For correlation analysis, we calculated individual AMI in the alpha band (8–13 Hz) for left and right sensors separately and then combined AMI over the sensors (see Zhigalov et al., 2019).

### 2.8 Sensor space data analysis

To assess coupling strength and latencies between the tagging signal and neuronal response, we computed the phase‐locking value (PLV; Lachaux, Rodriguez, Martinerie, & Varela, 1999). We applied the PLV to specifically assess phase relationship between the tagging signal and MEG, because the tagging signal by design was phase‐modulated with constant amplitude.

The signals were filtered using fourth‐order Butterworth zero‐phase filter implemented as combination of high‐pass and low‐pass filters with cut‐off frequencies 55 and 75 Hz, respectively, and PLV was computed as follows

(3) $$\mathrm{PLV}=\left|\frac{\sum e^{i\left(x-y\right)}}{N}\right|$$

where *x* and *y* are instantaneous phases of the filtered signals (i.e., MEG and tagging signal), *N* is the number of samples, and || denotes an absolute value. The instantaneous phase was estimated using Hilbert transform after band‐pass filtering. The bandwidth of the filter was slightly larger (55–75 Hz) than the bandwidth of the tagging signal (60–70 Hz) to avoid aliasing.

**FIGURE 2:**
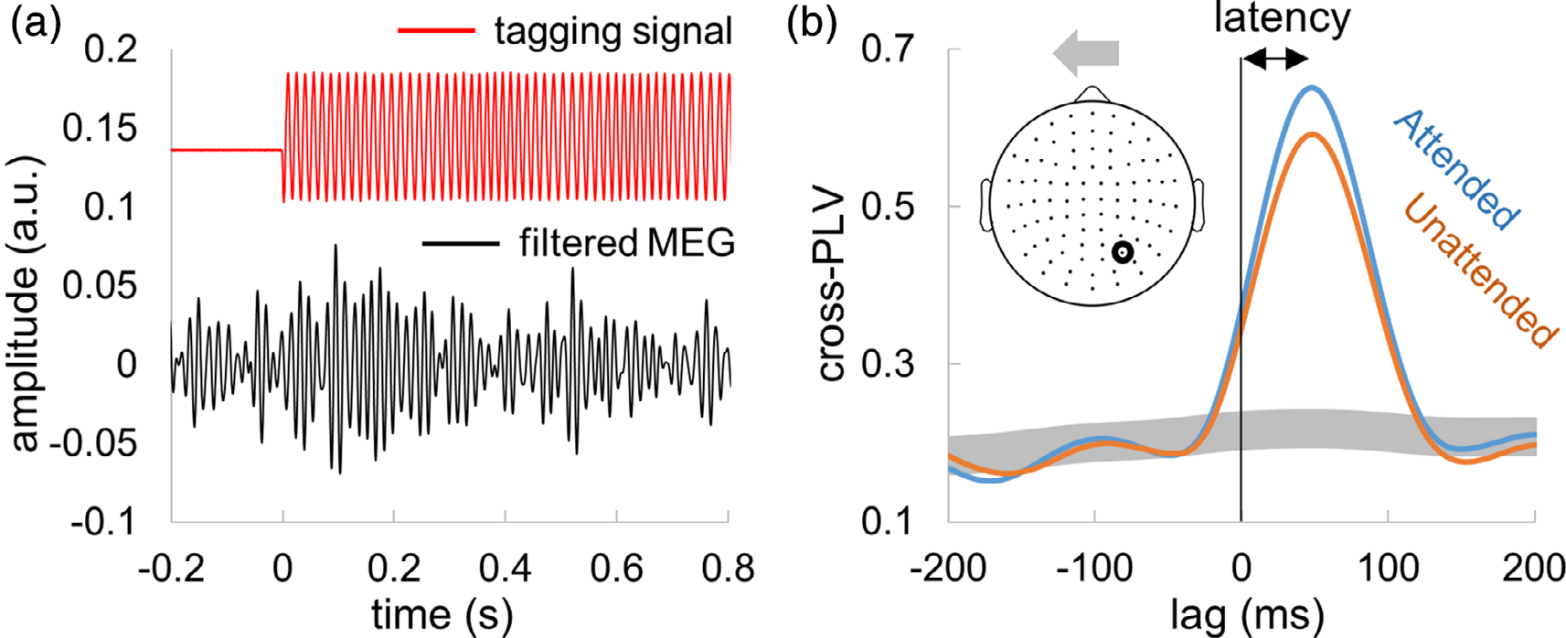
Estimation of response latency using the broadband frequency tagging technique. (a) Example of a single trial tagging visual signal (recorded by photodetector attached to the projector screen) and the magnetoencephalography (MEG) response. (b) Cross‐phase‐locking value (PLV) at multiple lags for a representative participant. The signals for attended compared to unattended objects showed larger cross‐PLV. The peak of the cross‐PLV reflects the delay in the MEG signals with respect to the visual input drive. Shaded area indicates 99% confidence interval

The PLVs were computed between the tagging signal and MEG signal (0–2 s from stimulus onset, Figure 2a) at multiple lags. The lags were chosen within range −200 to 200 ms (1 ms step) to fully capture the early neuronal response to the tagging signal. This is akin to calculating the cross‐correlation, where the correlation coefficient is replaced by PLV. These cross‐PLVs were estimated for each trial and averaged separately for trials comprising attended and unattended stimuli (Figure 2b). Then, maximum value of cross‐PLV and corresponding latency were assessed for each MEG sensor and condition.

To compensate for random fluctuations in the MEG at the higher frequencies, we computed the 99% confidence interval for the cross‐PLV (Figure 2b). The confidence interval was estimated from surrogate data that were generated by circularly shifting the MEG signal at random time points and subsequently computing cross‐PLV for 100 shifts. The maximum value of cross‐PLV was well above the 99% confidence interval for all the participants (see Supplementary Figure S2).

To assess differences between conditions (i.e., attended versus unattended stimuli) statistically, we utilized the pairwise *t* test.

### 2.9 Source space data analysis

AMI was computed in source space using dynamical imaging of coherent sources (DICS; Gross et al., 2001) spatial filter approach as implemented in Fieldtrip (Andersen, 2018; Oostenveld et al., 2011).

To build a forward model, we first manually aligned the MRI images to the head shape digitization points acquired with the Polhemus FASTRAK. Then, the MRI images were segmented, and a single shell head model was prepared using surface spherical harmonics fitting the brain surface (Nolte, 2003).

The time–frequency analysis (using multitaper frequency transformation as implemented in Fieldtrip) has been applied to the data in the interval 0–2 s after flickering stimuli onset. DICS spatial filters were computed for 10 mm grid where individual anatomy was warped into standard MNI template. Finally, AMI (see Equation (1)) was computed for each grid point at the alpha frequency (8–13 Hz) and the tagging response (55–75 Hz).

The difference between locations of sources showing maximum power at the alpha band and tagging response, was assessed by extracting the coordinates (along the interior‐superior z‐axis) of these sources for each participant separately, and then applied the *t* test over participants.

## 3 RESULTS

In this study, participants performed a cued visual attention task in which stimuli were frequency tagged using independent 60–70 Hz broadband signals in the left and right visual hemifield.

### 3.1 Behavioral performance

In this study, we used a relatively small number of trials (5%) for the invalid cue in order to maximize the attention effect driven by validly cued trials. This way, the behavioral responses in invalidly cued trials were too few for quantifying the behavioral effects. The hit rate was 75 ± 7% which was close to the expected 80% detection rate (see Supplementary Figure S1a). The false alarms (i.e., response to stimulus with invalid cue) were relatively low 2 ± 1% mainly explained by the low occurrence (5%) of invalid cues (see Supplementary Figure S1b).

### 3.2 Power of neuronal response modulated by spatial attention

**FIGURE 3:**
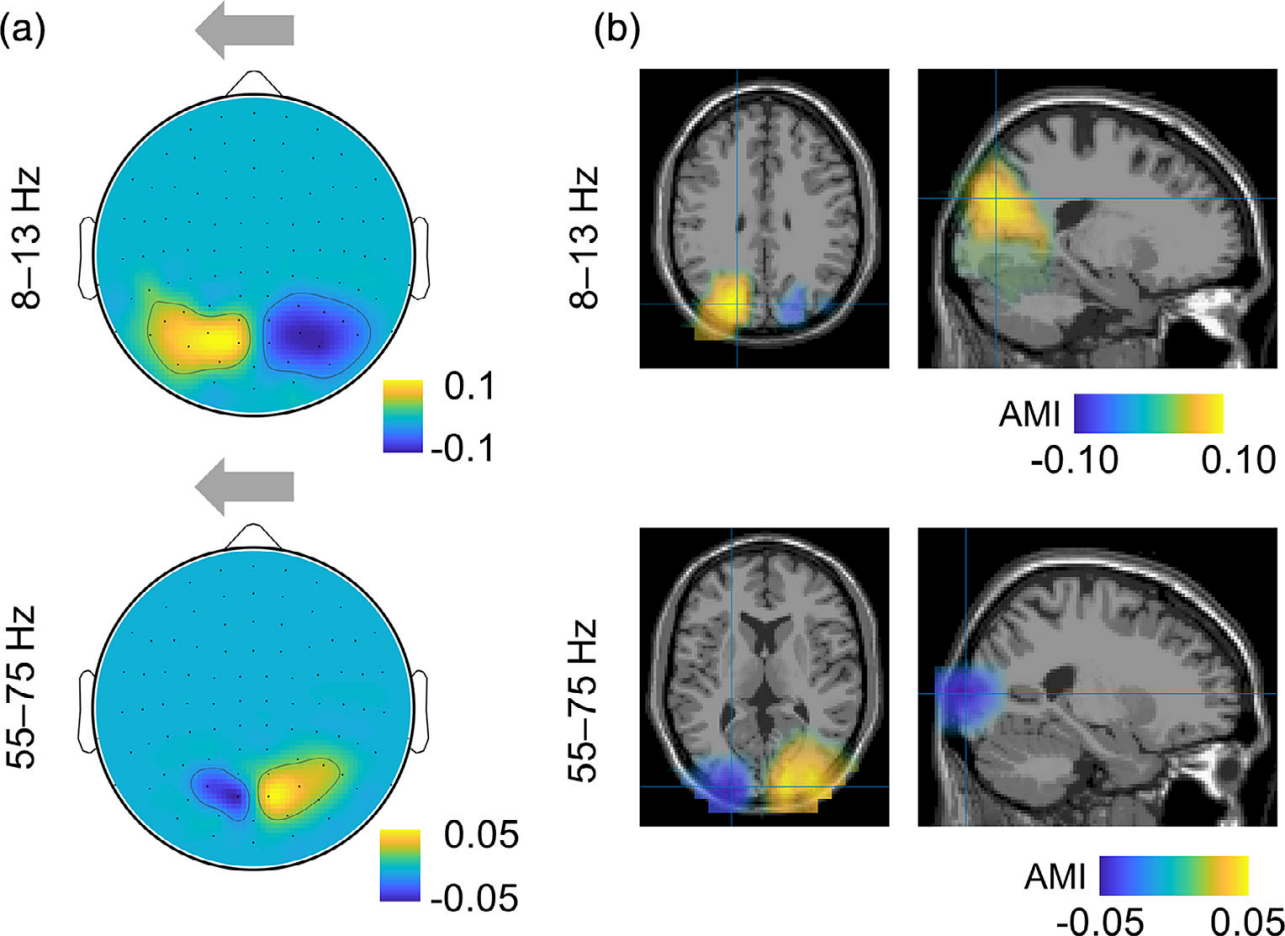
Attention modulates power of the alpha oscillations (8–13 Hz) and tagging response (55–75 Hz). The attention modulation index (AMI) was calculated by subtracting the estimated power for unattended (here, *attend right*) from attended (here, *attend left*) trials in the cue target interval (0–2 s). (a) Sensor‐level AMI for the alpha oscillations (top) and tagging response (bottom). (b) Source estimates of the AMI for the alpha (top) and tagging response (bottom). Note the sources around the parieto‐occipital sulcus for the alpha band modulation and early visual cortex for the tagging response

We first quantified the modulations of neuronal activity by calculating the time–frequency representation of power. Next, we calculated the AMI by subtracting the alpha power for attended from unattended trials in the cue‐target interval (0–2 s). The group level AMI was well in line with our previous observations for the alpha power and the tagging responses at 55–75 Hz (Figure 3a). The alpha power decreased contralaterally (and increased ipsilaterally) to the attended hemifield (~10%). An opposite pattern was observed at the tagging responses, where power of the tagging response increased contralaterally (and decreased ipsilaterally) to the attended hemifield (~5%). We applied a source modeling based on a beamforming approach to estimate the locations of the underlying neuronal sources. While the alpha power was localized in the parieto‐occipital cortex, the high frequency tagging response was generated in the primary visual cortex (Figure 3b). Interestingly, sources of neuronal response for even lower frequency tagging signals (i.e., 12 Hz) were also located in the primary visual areas (Parkkonen, Andersson, Hämäläinen, & Hari, 2008); although more recent study (Tabarelli, Keitel, Gross, & Baldauf, 2020) showed involvement of higher areas in visual hierarchy (e.g., ventral and dorsal streams) at certain stimulus frequencies.

### 3.3 Sources of alpha and tagging response are spatially distinct

**FIGURE 4:**
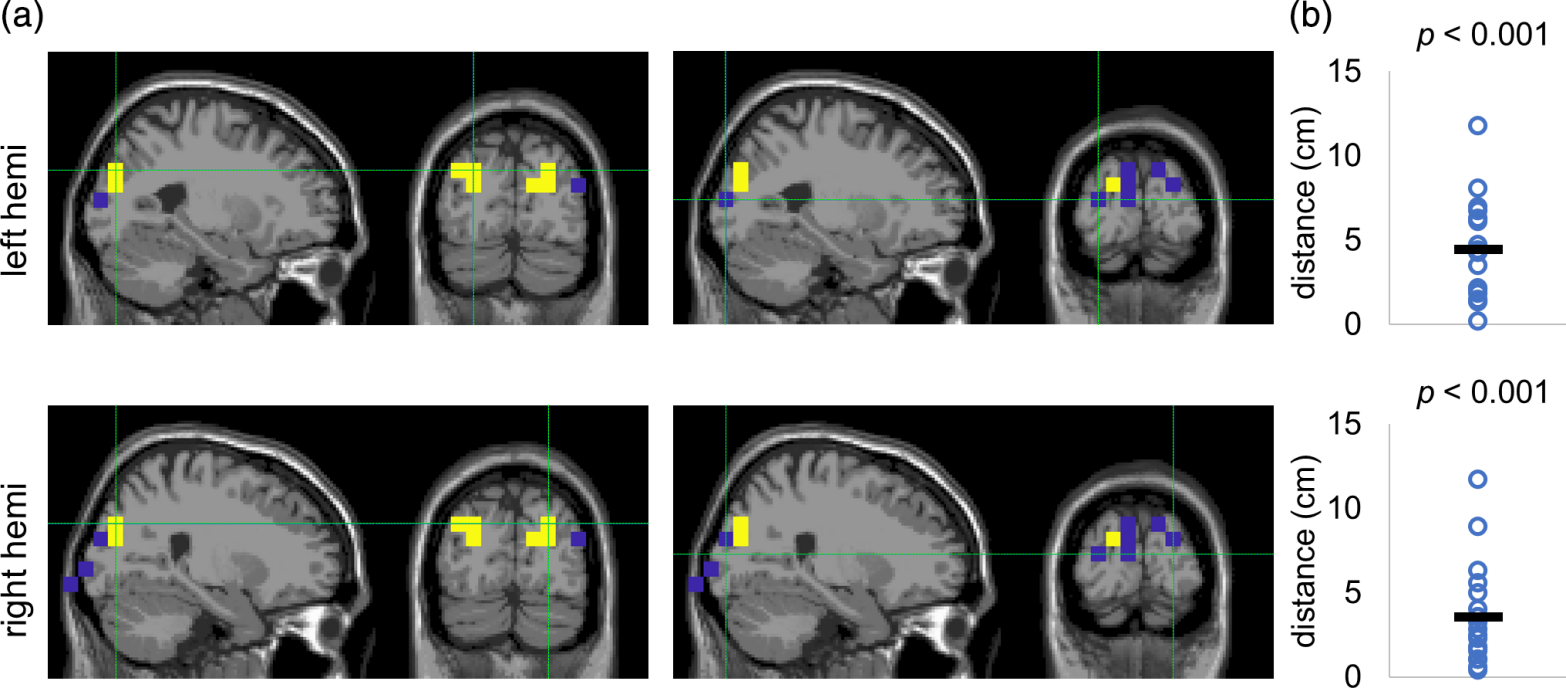
Source‐level attention modulation index (AMI) for the alpha and tagging response are spatially distinct. (a) The peak individual values of alpha AMI (yellow dots; left plots) were located around the parieto‐occipital cortex while largest individual values of tagging AMI (blue dots; right plots) were located closer to the primary visual cortex. (b) The distances between the peak locations of the AMI for the alpha and tagging response in individual subjects were significantly larger than zero in both hemispheres

It can be readily seen in both sensor‐ and source‐level analyses (Figure 3) that the spatial patterns for the alpha power and the tagging responses are distinct. Next, we assessed the individual distances between the source peaks of the alpha and frequency tagged response (Figure 4a). The yellow and blue markers indicate respectively the individual locations of the strongest alpha and frequency tagging modulations. Statistical analysis showed that the alpha modulation was on average about 4 cm anterior‐superior to the modulation of the tagging responses (*t*(17) = 6.63, *p* < .001 (left hemisphere) and *t*(17) = 5.33, *p* < .001 [right hemisphere], *t* test; Figure 4b). The effect was similar for both left and right hemispheres. While the sources of the tagging response were largely located in the primary visual cortex, sources of the alpha oscillations were located closer to posterior parietal cortex. Similar observations but without rigorous testing have been made in a spatial attention paradigm in the alpha and gamma frequencies (Bauer, Stenner, Friston, & Dolan, 2014). Our results suggest that sources of the alpha and tagging responses are spatially distinct and therefore likely to support different mechanisms.

### 3.4 Alpha power is not related to the power of tagging response

**FIGURE 5:**
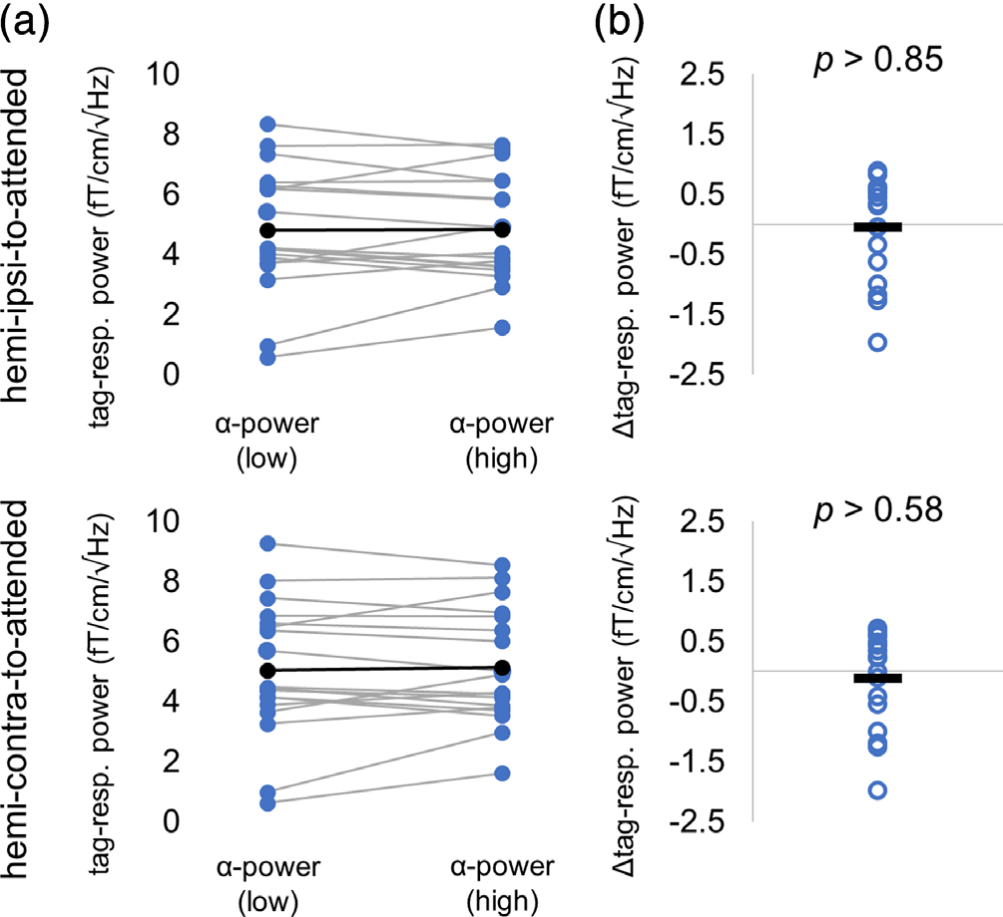
The tagging response power with respect to the median split of low and high alpha power over trials. (a) Power of the tagging response (denoted as tag‐resp. power) for individual subjects when pooled over trials with respect to low and high alpha power. (b) Pair‐wise difference of the data shown in (a). These findings demonstrate an absence in correlation between the alpha power and the frequency tagged signals

To further investigate the relationship between the alpha power and tagging response modulation, we computed—per participant—the power of the tagging response with respect to the median split of the trials according to alpha power (Figure 5a). The frequency tagging responses with respect to low and high alpha power were the averaged over participants and then compared using the pairwise *t* test (Figure 5b). Pairwise comparison revealed a complete absence of a relation (*t*(17) = −0.19, *p* > .85 (ipsilateral to attended) and *t*(17) = −0.57, *p* > .58 (contralateral to attended), pairwise *t* test). These results support the earlier notion that although the power of the alpha and tagging response are modulated by attention, they are not directly coupled.

### 3.5 Attention increases the magnitude of the tagged MEG response

**FIGURE 6:**
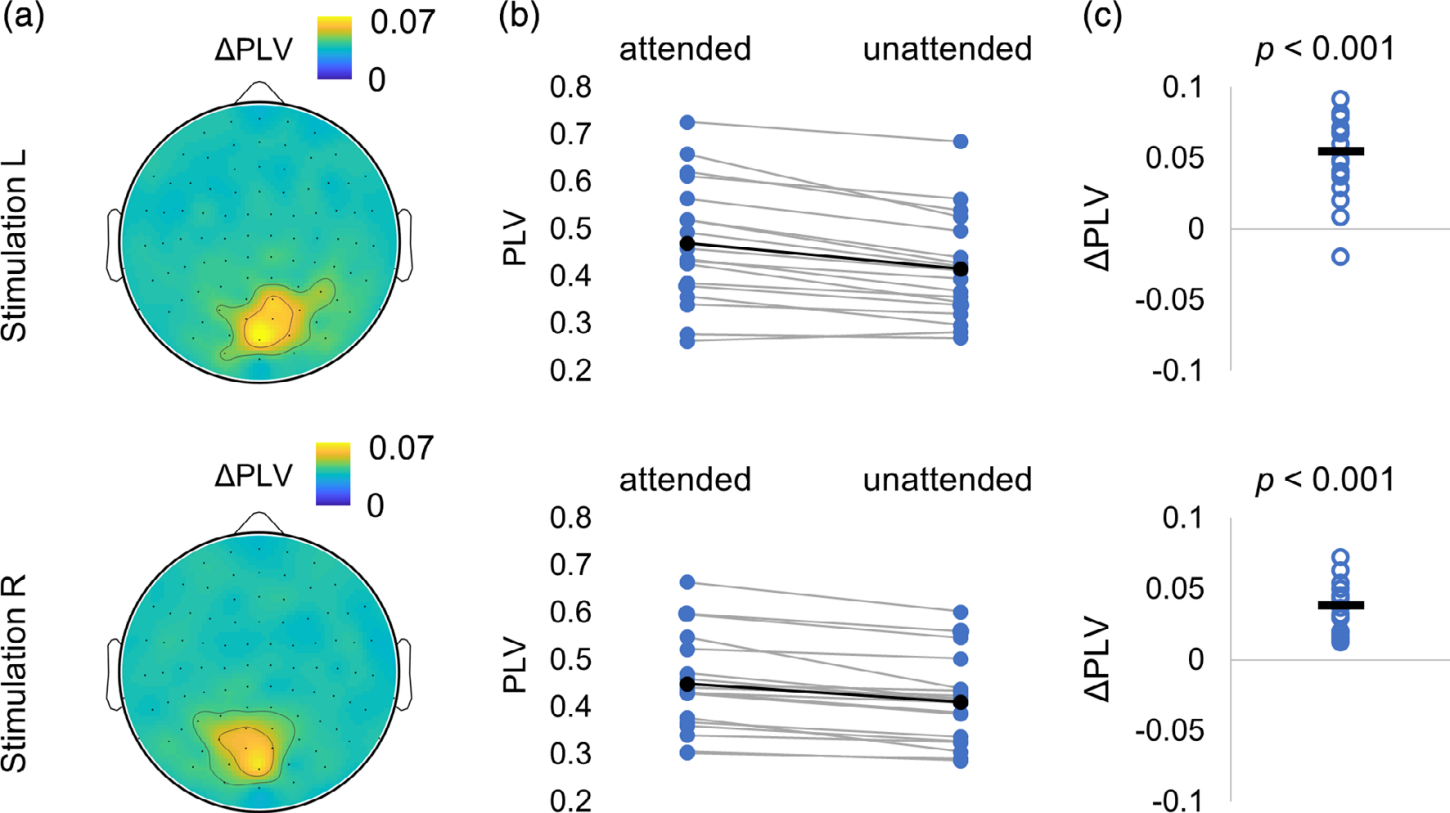
Attention modulates coupling strength between the tagging signal and MEG. (a) Spatial patterns (topographies) of the cross‐phase‐locking values (cross‐PLV) at 45–60 ms between the visual drive (photo diode) and the MEG signal at group level for attended and unattended stimuli for the stimulation on the left (top panel) and on the right (bottom panel). The cross‐PLV was derived in the 0–2 s interval after stimulation onset and was averaged over planar gradiometers. (b,c) Pairwise comparison showed a significant increase in maximum cross‐PLV (latency 45–60 ms) for attended versus unattended objects

Similarly to the power of tagging response at 55–75 Hz (Figure 3a), the cross‐PLV at lags of 45–60 ms (see methods, Figure 2b) were larger for attended compared to unattended stimuli in the occipital sensors. To assess the difference statistically, we selected the strongest responding sensor (planar gradiometers) when considering the combined conditions for each participant. We observed significant differences in the cross‐PLV measure between tagging and MEG signals (Figure 6) related to attention (*t*(17) = 6.51, *p* < .001 [right sensors] and *t*(17) = 6.68, *p* < .001 [left sensors], paired *t* test). These results are in line with earlier findings (Zhigalov et al., 2019) as well as Figure 3 showing that attention increases the magnitude of the tagged response.

### 3.6 Attention does not affect latency of the tagging MEG response

**FIGURE 7:**
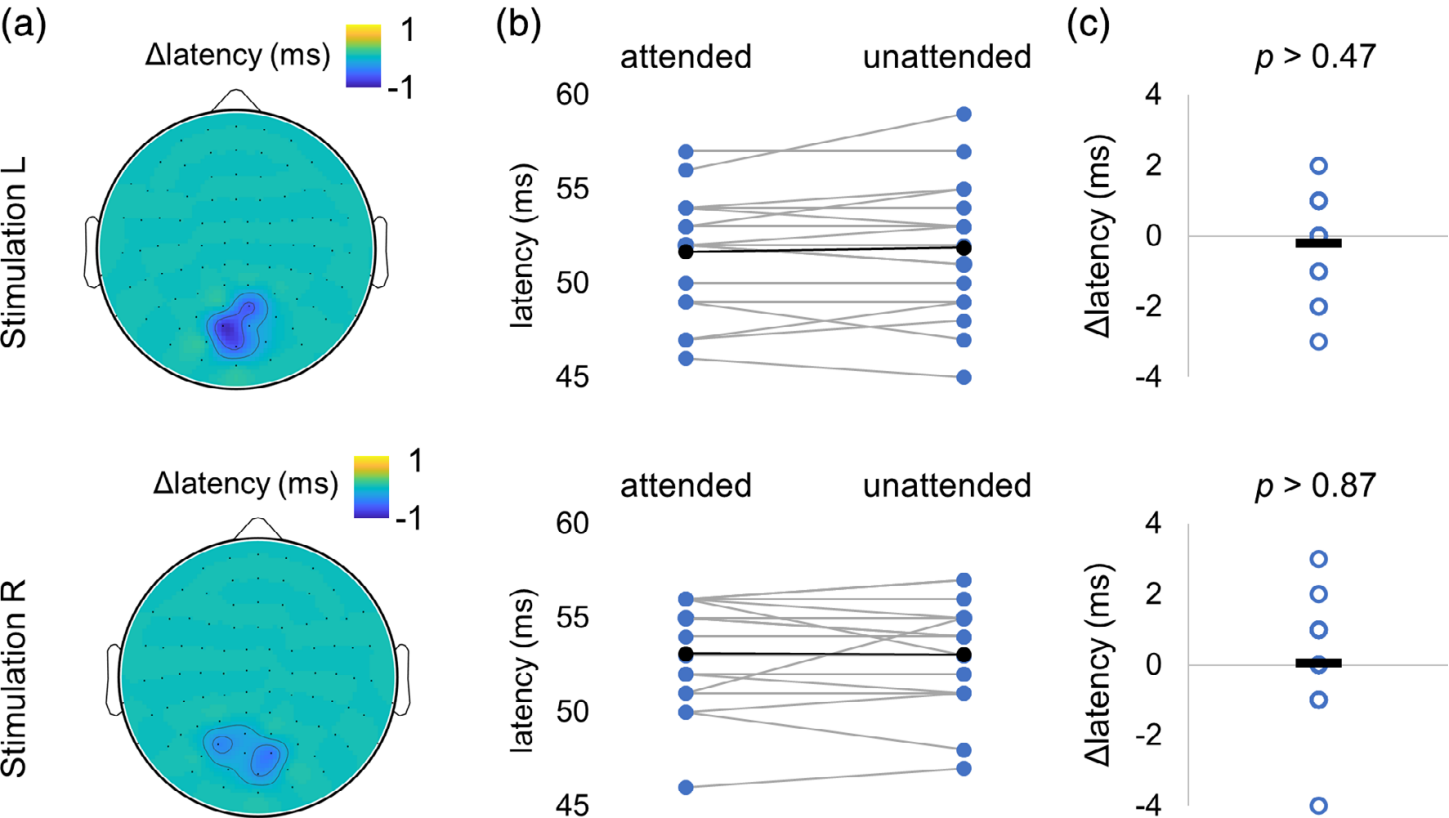
Attention did not affect the neuronal response latency between tagging signal and MEG. (a) Group level spatial patterns of latencies for attended and unattended stimuli for the stimulation on the left (top panel) and on the right (bottom panel). (b,c) Pairwise comparison did not show any significant changes in latencies for attended versus unattended objects

To assess the latency between the visual input and the MEG response, we estimated the lag associated the maximum cross‐PLV (see methods, Figure 2b). We found that attention did not affect the latency between the neuronal response and the tagging signal in the occipital sensors of interest (Figure 7a). For statistical comparison, we considered the strongest responding sensor left and right sensors for each participant, in the same manner as it has been done for PLV (Figure 6b). The results did not show a robust differences (*t*(17) = −0.75, *p* > .47 (right sensors) and *t*(17) = 0.16, *p* > .87 (left sensors), paired *t* test) in neuronal response latencies with attention (Figure 7b,c).

### 3.7 Relationship between power of ongoing alpha activity and parameters of tagging response

**FIGURE 8:**
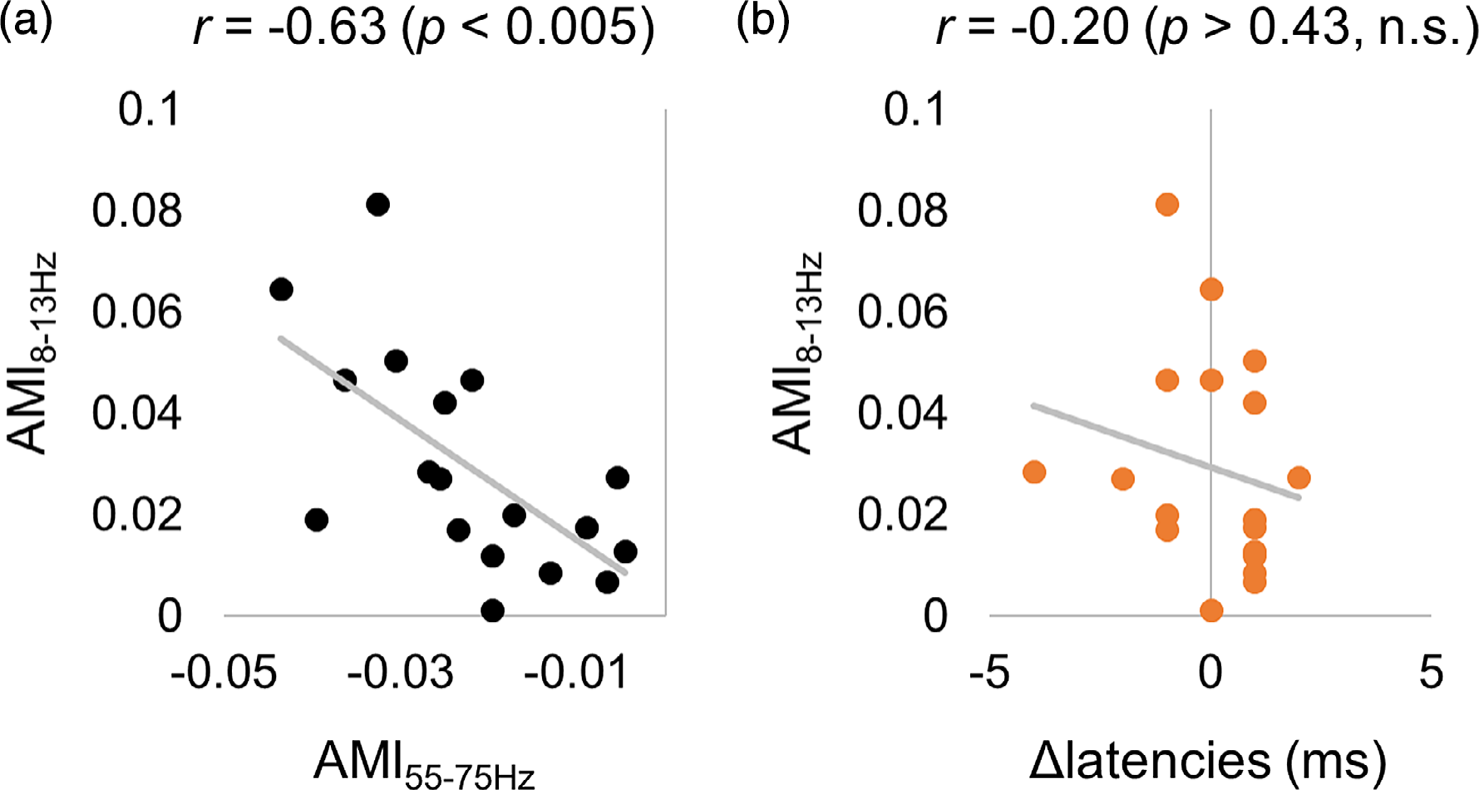
Relationship between attentional modulation of the alpha power and the tagging response over subjects. (a) The decrease in alpha power with attention was correlated with an increase in the tagging response with attention over subjects. (b) Attentional modulation of the alpha power was not related to attentional modulation of the response latency over subjects

We assessed the relationship between individual AMI at the alpha band and AMI of tagging response, and also between the individual AMI at the alpha band and response latencies. First, we computed the correlation between individual AMI at the alpha frequency and tagging response at 55–75 Hz (Figure 8a). These results showed a negative correlation (*r* = −.63 [Spearman correlation], *p* < .005) between the modulation of power at the alpha and tagging responses, which is in line with earlier observations (Zhigalov et al., 2019). Similarly, we correlated individual AMI in the alpha band and the response latencies (Figure 8b); however, the correlation was not significant (*r* = −.20 [Spearman correlation], *p* > .43).

## 4 DISCUSSION

In this MEG study, we used a spatial attention task in combination with a novel broadband frequency tagging technique. We found that the power of alpha activity and the tagging response were modulated by attention, confirming earlier results (Zhigalov et al., 2019). Source modeling of the MEG data allowed us to identify the neuronal generators of the frequency tagging signal in early visual regions, and the alpha oscillations generators around parieto‐occipital sulcus. Importantly we showed that the power of alpha and tagging response was not related at single trial level. By further analyzing the broadband tagging response, we showed that the response delays were not modulated by attention.

### 4.1 Neuronal excitability and alpha change with attention

Numerous studies have demonstrated that alpha oscillations are top‐down modulated when attention is allocated (Foxe & Snyder, 2011; Klimesch, 2012; Müller & Weisz, 2012). This has resulted in the idea that alpha oscillations serve to control neuronal gain in early visual regions (Jensen, Bonnefond, Marshall, & Tiesinga, 2015; Jensen & Mazaheri, 2010; Spaak, Bonnefond, Maier, Leopold, & Jensen, 2012). Using multicontact laminar electrodes to measure spontaneous signals simultaneously from all layers of V1, Spaak et al. (2012) found a robust coupling between alpha phase in the deeper layers and gamma amplitude in granular and superficial layers. In the same vein, a study by Jensen et al. (2015) proposed layer‐ and frequency‐specific mechanism of feedforward and feedback visual processing, in which alpha oscillations could modulate gamma activity in V1 area. We here operate under the premise that neuronal excitability and gain control can be quantified by means of the frequency tagged response.

**FIGURE 9:**
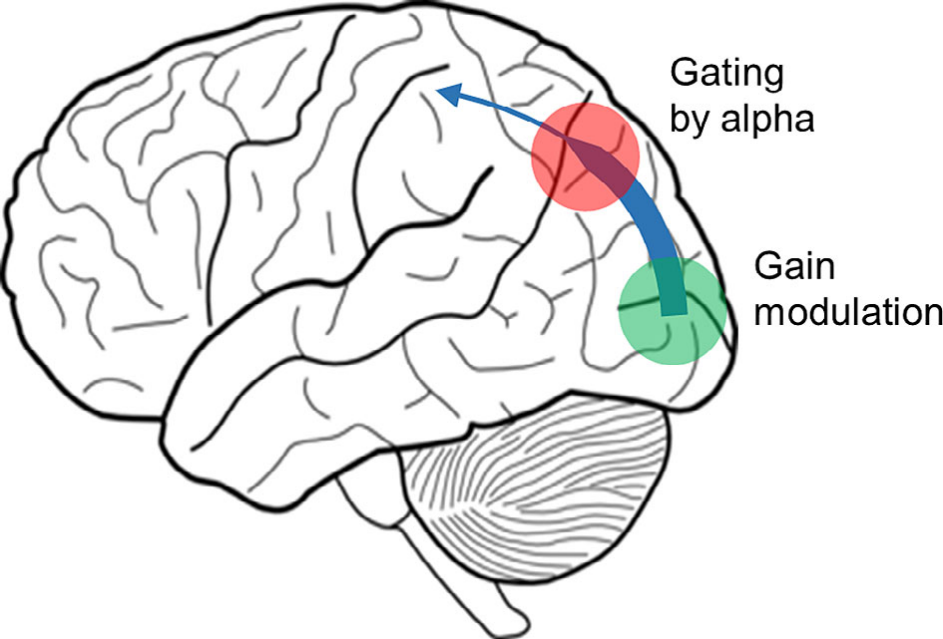
We find that spatial attention modulates the frequency tagging response in early visual regions (green) and alpha oscillations around the parieto‐occipital sulcus (red). Importantly the alpha activity and the frequency tagging response were not correlated over trials. This points to a scheme in which alpha oscillations gate the information flow in downstream visual regions without directly controlling the gain in early visual regions

Our findings challenge the notion that alpha oscillations exert gain control in early sensory regions. First, the power at the alpha and tagging responses were not correlated at the single trial level as also demonstrated in previous findings (Antonov, Chakravarthi, & Andersen, 2020; Gundlach et al., 2020; Zhigalov et al., 2019). Second, the sources of the alpha oscillations were localized around the parieto‐occipital sulcus while the sources of tagging response were located in primary visual cortex. We conclude that alpha oscillations do not serve to adjust the gain in early visual regions; rather the alpha oscillations serve to gate neuronal activity in regions downstream of the visual cortex. As shown in Figure 9, we propose that this gating serves the allocation of neurocomputational resources and modulates the feedforward flow in parieto‐occipital areas. As proposed in previous work (Jensen & Mazaheri, 2010), the gating could be implemented neurophysiologically by GABAergic pulsed inhibition in the alpha frequency range that will limit neuronal processing and the feedforward flow.

Our results are in line with recent studies utilizing a similar cuing attention paradigm when tagging at lower frequencies (Antonov et al., 2020; Gundlach et al., 2020; Keitel et al., 2019). In general, these studies show that the magnitude of the alpha‐band activity does not modulate steady‐state visual evoked potentials (SSVEP). Hence, the attentional dynamics of early sensory gain control and alpha‐band oscillations may represent two complementary mechanisms of spatial attention. In a study, Keitel et al. (2019) examined modulations of SSVEPs for stimuli that flickered in the alpha band. Evoked SSVEP signals increased for the attended versus unattended stimulus, whereas the opposite pattern was observed for induced alpha‐band oscillations, although both signals overlapped in the frequency domain. The absence of comodulation of the signals supports the notion that modulations of SSVEPs and alpha oscillations reflect different mechanisms. These findings are interesting in the light of papers arguing for entrainment of alpha oscillations in attention tasks (Spaak, de Lange, & Jensen, 2014). A study by Gundlach et al. (2020) complement and expand these findings by introducing an unbiased estimation of the attentional modulation of alpha oscillations and SSVEPs (above 14 Hz) with an experimentally controlled baseline. Importantly, they found that SSVEPs and alpha power were not correlated over trials. Finally, a study by Antonov et al. (2020) show enhanced gain for attended stimuli, as indexed by SSVEP amplitudes, rather than suppressed gain of unattended stimuli that was not preceded by changes in the alpha band. This result further suggests that alpha oscillations do not reflect direct gain control.

Despite the similarities with these studies in terms of experimental paradigm, our experimental approach has several advantages. First, we used the novel high frequency tagging technique (60–70 Hz) that produces invisible flicker and thus, does not disrupt ongoing alpha oscillations, which in turn allows us to better dissociate frequency specific effects of attention. Second, we used source reconstruction on the MEG signals to localize of the generators of the alpha oscillations and tagging response which provide a better spatial resolution compared to EEG.

### 4.2 Attention does not modulate latencies of the tagging response

It is debated to what extend attention modulates the speed of visual processing. Several electrophysiological studies in monkeys (Lee & Maunsell, 2010; Lee, Williford, & Maunsell, 2007) showed that contrast but not attention modulated the latency of the response. However, recent studies (Galashan et al., 2013; Sundberg et al., 2012) showed that attention impacted the latencies of neuronal responses in V4 and MT areas, respectively, although the effect was relatively small (1–2 ms). Using broadband frequency tagging, we estimated the delay in neuronal activation with respect to the visual input by time‐shifting the two signals in order to identify when they were strongest coupled. Consistent with the literature (e.g., Lee & Maunsell, 2010; Maunsell, 2015) we found that the visual cortex responded maximally ~50 ms after the visual input; however, attention did not modulate the response latencies. Consistent with prevailing views (Tallon‐Baudry, 2012) we conclude that spatial attention does not modulate response latencies in early visual cortex; however, our approach does provide an exciting new tool for estimating the delay of neuronal activation in visual cortex.

### 4.3 Power of the response but not latency of the tagging response is affected by ongoing alpha activity at group level

We replicated earlier observations demonstrating that attentional modulation of power of the alpha and tagging responses are negatively related at group level (Zhigalov et al., 2019). However, there was no evidence that the alpha power was correlated with the response latencies. The overparticipant correlation does point to a relation between neuronal excitability in early visual regions and alpha power; however, this effect might be partly explained by different signal‐to‐noise ratios in the participants and thus not functional.

It should also be noted that such correlation across participants was not significant in a similar study (Keitel et al., 2019). One plausible explanation is the difference in frequencies of the tagging signals: while we used stimulation at 60–70 Hz, the study by Keitel et al. used tagging frequencies within the alpha band (10 and 12 Hz).

## 5 CONCLUSION

We conclude that alpha oscillations are not directly involved in gain control of neuronal activity in early visual regions. Rather the alpha oscillations might serve to gate the feed‐forward flow in downstream regions, for example, around the parieto‐occipital sulcus. In future work, it would be of great interest to uncover the neuronal mechanisms implementing the gating in relation to the alpha oscillations as well as the gain control as reflected by the rapid frequency tagging responses.

## Supporting information


**Supplementary Figure S1** Behavioral results for individual participants. (A) Hit rate was close to the expected 80% detection rate. (B) False alarms (i.e., response to stimulus with invalid cue) were extremely low because to small number of catch trials (5%).

**Supplementary Figure S2** Cross‐PLV for individual participants. Black and red lines indicate cross‐PLV averaged over trials with “attended” and “unattended” stimuli, respectively. Cross‐PLVs was computed for an occipital sensor with strongest response to the tagging signal. Shaded area indicates 99% confidence interval.

## Data Availability

Data and code are available from the corresponding author upon reasonable request.
