# Supplementary figures and images for: Alpha oscillations do not implement gain control in early visual cortex but rather gating in parieto‐occipital regions
Source: Hum Brain Mapp. 2020 Aug 21;41(18):5176–86. doi: 10.1002/hbm.25183 (PMC7670647; doi:10.1002/hbm.25183)

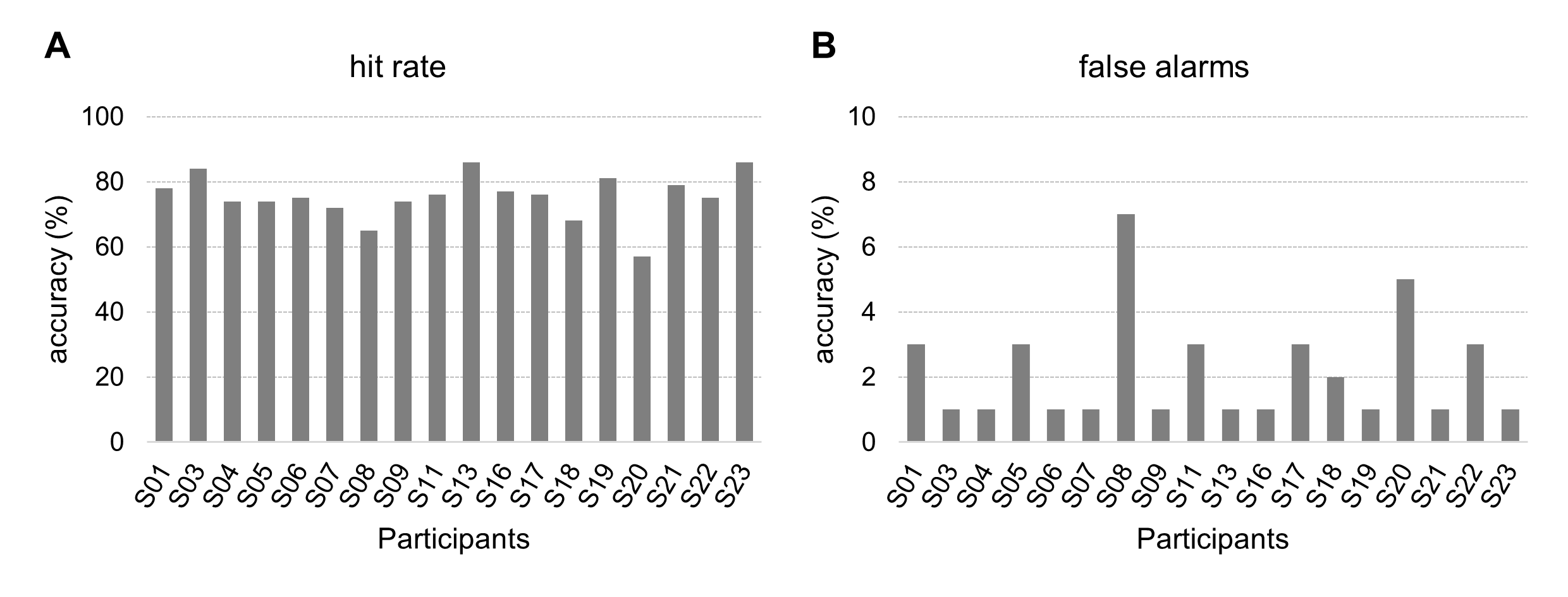

Supplement: Supplementary file 1 — Supplementary Figure S1 Behavioral results for individual participants. (A) Hit rate was close to the expected 80% detection rate. (B) False alarms (i.e., response to stimulus with invalid cue) were extremely low because to small number of catch trials (5%). [file HBM-41-5176-s001.tif]

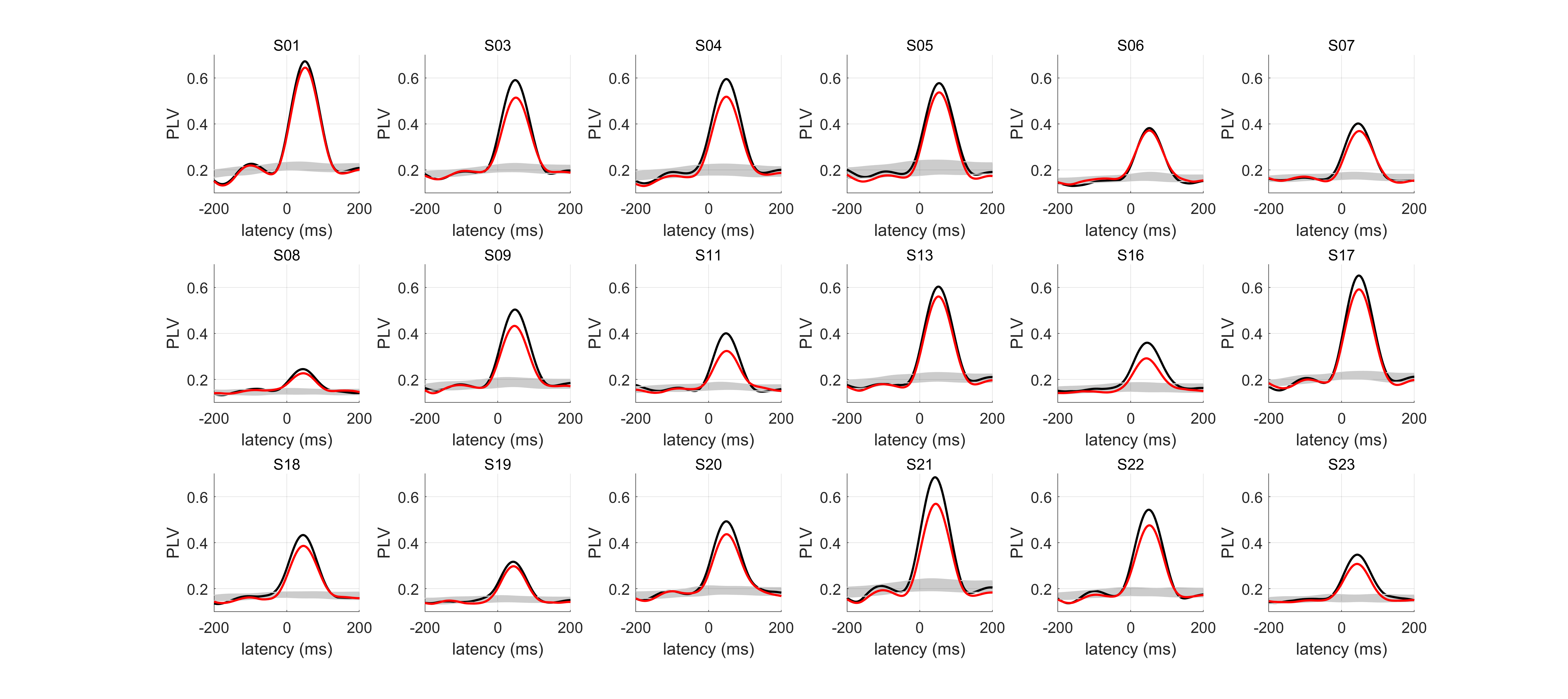

Supplement: Supplementary file 2 — Supplementary Figure S2 Cross‐PLV for individual participants. Black and red lines indicate cross‐PLV averaged over trials with “attended” and “unattended” stimuli, respectively. Cross‐PLVs was computed for an occipital sensor with strongest response to the tagging signal. Shaded area indicates 99% confidence interval. [file HBM-41-5176-s002.tif]
